# Early parasympathetic dysfunction in Parkinson’s disease: insights from information-theoretic analysis

**DOI:** 10.3389/fnetp.2025.1680069

**Published:** 2025-09-26

**Authors:** Jana Cernanova Krohova, Jana Oleksakova, Zuzana Turianikova, Barbora Czippelova, Milan Grofik, Egon Kurca, Michal Javorka

**Affiliations:** ^1^ Department of Physiology and Biomedical Centre Martin (BioMed Martin), Jessenius Faculty of Medicine in Martin, Comenius University Bratislava, Martin, Slovakia; ^2^ Clinic of Neurology, Jessenius Faculty of Medicine in Martin, Comenius University Bratislava, Martin, Slovakia

**Keywords:** parasympathetic nervous system, respiratory sinus arrhythmia, respiratory heart rate variability, Parkinson’s disease, network physiology, information-theoretic analysis, constipation

## Abstract

**Introduction:**

Parasympathetic nervous system (PNS) dysfunction in Parkinson’s disease (PD) has been frequently evaluated using heart rate variability (HRV) analysis in the time and frequency domains. Findings across studies have been inconsistent, limiting a unified understanding of early autonomic impairment.

**Methods:**

In this study, we applied both conventional and advanced analytical methods to evaluate cardiovascular PNS function in the early-stage PD patients. Sixteen individuals with PD (<6 months after motor signs occurrence) and sixteen age- and sex-matched healthy controls were assessed across three protocol phases (supine rest, head-up tilt, and supine recovery). Traditional HRV analysis in the high-frequency band was used to estimate the overall respiratory heart rate variability (RespHRV; updated and more appropriate term for the respiration-related heart rate oscillations formerly called respiratory sinus arrhythmia, RSA) magnitude. To distinguish between baroreflex-mediated and non-baroreflex RespHRV mechanisms, we employed multiscale Partial Information Decomposition (PID), an information-theoretic method. Cardiac baroreflex sensitivity (BRS), reflecting reflex parasympathetic control, was assessed using a causal estimation approach, further supported by a PID-derived parameter quantifying coupling between systolic arterial pressure and R-R intervals. Additionally, the presence of constipation – a clinically relevant non-motor symptom indicative of parasympathetic dysfunction was used to stratify the PD cohort.

**Results:**

Early-stage PD patients exhibited signs of parasympathetic impairment, particularly during orthostatic stress. HRV analysis showed reduced HF power during head-up tilt, while causal BRS was significantly lower across all protocol phases in the PD group. PID analysis further demonstrated a significant reduction in baroreflex-mediated mechanism of RespHRV during head-up tilt in PD patients compared with healthy controls, indicating early dysfunction of the cardiac chronotropic baroreflex. This impairment was more pronounced in the group with gastrointestinal issues (with the presence of constipation).

**Discussion:**

These findings support the α-Synuclein Origin site and Connectome model, which proposes that PD patients whose neurodegeneration originates in the peripheral autonomic nervous system are characterized by early and more severe autonomic dysfunction.

## 1 Introduction

Parkinson’s disease (PD) is a progressive neurodegenerative disorder characterized not only by motor symptoms (resting tremor, rigidity, hypokinesia, and postural instability) but also involves significant autonomic nervous system dysfunction, affecting both the sympathetic and parasympathetic nervous system (PNS) (Sabino-Carvalho et al., 2021). Autonomic dysfunction in PD affects various organ systems and functions, resulting in cardiovascular abnormalities (orthostatic hypotension, supine hypertension, absence of nocturnal blood pressure decrease), gastrointestinal issues (constipation), urological, sexual, and thermoregulatory dysfunctions (Pfeiffer, 2020). Despite growing recognition of these non-motor manifestations potentially preceding the onset of motor symptoms by years, clinical diagnosis of PD is still primarily based on motor symptoms (Chaudhuri et al., 2006). Autonomic dysfunction in PD is believed to result from the accumulation of abnormal α-synuclein protein in both central and peripheral components of the autonomic nervous system. These pathological deposits, known as Lewy bodies, are commonly found in autonomic control centres, including the hypothalamus, sympathetic (e.g., intermediolateral nucleus of the thoracic cord and sympathetic ganglia), and parasympathetic regions of brainstem (e.g., dorsal motor nucleus of the vagus nerve, nucleus ambiguus), potentially disrupting autonomic control, including reflexes (Braak et al., 2003; Wang et al., 2020).

According to the current knowledge about the pathogenesis of PD, it is possible to distinguish two subtypes of PD (Borghammer, 2021) based on the anatomical origin of neurodegeneration: the “body-first” and “brain-first” subtypes. In the “body-first” subtype of PD, pathology originates in the peripheral nervous system and is presumably associated with a more pronounced impairment of the autonomic nervous system, including symptoms such as constipation. In contrast, in the “brain-first” subtype of PD initial α-synuclein pathology occurs in the central nervous system. This form is typically associated with slower disease progression and fewer early autonomic symptoms, typically without the presence of constipation (Velucci et al., 2025).

Cardiac PNS dysfunction in PD remains relatively underexplored, largely due to methodological limitations in its evaluation. Non-invasive studies evaluating the cardiovascular autonomic control in PD patients were primarily focused on heart rate variability (HRV) analysis in frequency and time domain. The high-frequency (HF) component of HRV, reflecting respiratory heart rate variability (RespHRV; referred to as respiratory sinus arrhythmia, RSA), serves as a well-established indicator of parasympathetic activity. In accordance with recent international expert recommendations, we use the updated term RespHRV, as it more accurately describes the physiological modulation of heart rate by respiration and avoids the misleading implication of a pathological arrhythmia inherent in the term RSA (Menuet et al., 2025). Although several studies reported a reduced HF power in patients with longer duration of PD (more than 2 years) suggesting diminished vagal activity (Li et al., 2021; Sabino-Carvalho et al., 2018; Soares et al., 2013) other studies did not confirm this finding (Oka et al., 2011; Vianna et al., 2016; Ke et al., 2017). In addition to HRV analysis employing RespHRV magnitude as a basic cardiovascular PNS activity index, baroreflex sensitivity (BRS) of the cardiac chronotropic arm – an index of reflex parasympathetic control (Eckberg and Sleight, 1992; van de Vooren et al., 2007; Carrasco-Sosa et al., 2005) – has also been found to be decreased in PD patients with a longer duration of the disease (Oka et al., 2011; Sabino-Carvalho et al., 2020), but in other studies no significant differences between control subjects and PD patients were found (Barbic et al., 2007; Friedrich et al., 2008).

Thus, considering the divergent results and a limited use of advanced methods in the non-invasive evaluation of PNS activity, the aim of this study was to assess changes in PNS function in the early stage of PD by applying both traditional and more advanced analytical approaches to address the various aspects of PNS impairment and to evaluate the potential of these methods in detecting early autonomic dysfunction.

Specifically, to evaluate the overall magnitude of RespHRV, we employed conventional HRV analysis in the HF band. Additionally, using the information-theoretic method — multiscale Partial Information Decomposition (PID) (Faes et al., 2018; Faes et al., 2017; Williams and Beer, 2010) – we were able to dissect baroreflex and non-baroreflex mechanisms contributing to the origin of RespHRV and compare their relative influence. The overall non-invasive assessment of PNS function was further complemented by evaluating the sensitivity and effectiveness of the cardiac chronotropic baroreflex arm, which primarily reflects reflex parasympathetic control. This was achieved using a more precise causal method of evaluation of BRS in a combination with PID-derived parameter characterizing the strength of coupling between systolic blood pressure and heart rate. Analyses were conducted using a three-phase protocol – supine rest, head-up tilt, and supine recovery – commonly applied in autonomic research (Thijs et al., 2021; Toska and Walloe, 2002). Supine rest provides a baseline for vagal activity, head-up tilt elicits cardiac parasympathetic inhibition and sympathetic activation directed to the heart and vasculature, and the return to supine captures parasympathetic reactivation during recovery. This dynamic sequence moves beyond static measures, enabling detection of subtle, state-dependent alterations in autonomic regulation that may otherwise remain hidden in PD.

Additionally, the presence of constipation – a clinically relevant non-motor symptom indicative of parasympathetic dysfunction and a potential early marker of PD “body-first” subtype – was used to stratify the PD cohort. We hypothesized that parameters reflecting PNS activity would be more impaired in PD patients with the presence of constipation – confirming the concept of more severe ANS dysfunction in the “body-first” subtype of PD.

## 2 Methods

This study included 16 patients in the early-stage of PD (8 women, mean age: 57.1 ± 8.6 (SD) years) and 16 age- and sex-matched healthy controls (8 women, mean age: 54.5 ± 8.4 years), with no significant difference in age between groups (P = 0.385). PD was clinically diagnosed according to the Movement Disorder Society (MDS) 2015 criteria, with the early-stage defined as the onset of motor symptoms within 6 months before the examination. Among the enrolled patients, 15 were receiving antiparkinsonian treatment (10 were treated with dopamine agonists, 5 with levodopa, and 4 with rasagiline), while 1 was untreated. To exclude short-term effects of pharmacological treatment on autonomic nervous system activity, patients on dopamine agonists or rasagiline discontinued their treatment 24 h before the examination, whereas those on levodopa paused treatment for 12 h. Exclusion criteria included obesity (body mass index (BMI) > 30 kg/m^2^, the mean BMI was 28.4 ± 5.6 kg/m^2^ in the PD group and 25.4 ± 4.6 kg/m^2^ in the C group, with no significant difference between groups P = 0.109), a history of diabetes mellitus, neuropathy, cardiovascular disease (ischemic heart disease, arterial hypertension, arrhythmias), use of medications affecting the autonomic nervous system, and regular intensive physical training. The presence of constipation in PD patients was determined by medical records or the prescription of medications used to treat this condition. Accordingly, PD group was divided into subgroups with or without this gastrointestinal symptom of dysregulation (GIT+ (n = 5, 4 women, mean age: 61.7 ± 3.5 years) and GIT- (n = 11, 4 women, mean age: 55.1 ± 9.5 years) subgroups, respectively). No significant difference between subgroups in age was found (P = 0.193). All procedures were approved by the Ethics Committee of the Jessenius Faculty of Medicine, Comenius University Bratislava (approval no. EK 78/2020; revised EK 75/2024) and complied with the ethical standards of the 1964 Declaration of Helsinki and its subsequent amendments. All participants provided written informed consent before participation.

### 2.1 Study protocol and data acquisition

The initial examination of the probands included collection of their medical history, a clinical neurological examination and anthropometric measurements. It was followed by a continuous non-invasive beat-to-beat recording of cardiovascular and respiratory parameters at a sampling frequency of 1000 Hz using a PowerLab 8/35 device (ADIntruments Pty Ltd, Australia). During whole examination protocol probands were positioned on the motor driven tilt table (Electric tilt table model 900-00, CNSystems, Austria) with their feet in contact with the footboard at the end of the table and restraining strap secured at the thighs level to ensure support and safety. The study protocol consisted of three phases: supine rest (15 min), head-up tilt (HUT, the subject was tilted to 45° on a tilt table for 8 min to induce mild orthostatic stress), and supine recovery (10 min). Throughout the protocol, we continuously and non-invasively recorded beat-to-beat R-R interval values (RR signal) using electrocardiography (CardioFax ECG-9620, Nihon Kohden, Japan). Systolic blood pressure (SAP) values for each heart beat were measured using the volume-clamp photoplethysmographic method (Finometer Pro, FMS, Netherlands), and respiratory volume (RESP signal) was recorded via respiratory inductive plethysmography (RespiTrace, NIMS, United States). For each breath, respiratory rate and tidal volume were measured from the calibrated RESP signal. Tidal volume was defined as the difference between local minimum (onset of inspiration) and subsequent maximum (offset of expiration). Respiratory rate in Hertz (Hz) was calculated as the reciprocal of the breathing cycle duration, defined as the time interval between two consecutive onsets of inspiration. All measurements were carried out in the morning hours between 8 and 11 a.m.

### 2.2 Data analysis

Stationary 300-beat segments used for further analysis were extracted as follows: for the supine rest phase, the analysed segment started 8 min after the beginning of this phase, for the HUT phase 3 min after the change of position, for the supine recovery phase 7 min before the end of this protocol.

Traditionally used spectral analysis of HRV was performed using the fast Fourier transform to obtain spectral power in the HF band (0.15–0.5 Hz). The RR time series was first resampled at 2 Hz using cubic spline interpolation to obtain an equidistant signal. Slower oscillations and trends were subsequently removed using the detrending method of Tarvainen et al. (2002). The mean power spectrum of each analysed segment was then computed, and spectral power in the HF band was quantified by integration. To assess BRS, we applied causal analysis of systolic blood pressure and heart rate in the frequency domain, focusing on the low-frequency (LF) band (0.04–0.15 Hz). A method based on a bivariate autoregressive model was used to estimate the unidirectional causal gain from SAP to RR, representing BRS. Causal gain values were calculated as the arithmetic mean of the gain values within the LF band and expressed as the magnitude of the RR interval response to a unit change in SAP of 1 mmHg (Porta et al., 2002). Model identification was performed using vector least squares estimation, with model order determined by the Akaike Information Criterion (Akaike, 1974).

In this study, we also employed PID (Faes et al., 2017; Williams and Beer, 2010) to decompose the mutual information shared between a target variable (RR signal) and two source variables (RESP and SAP) into separate unique (transfer entropies (TEs) SAP→RR and RESP→RR), redundant (TE from RESP through SAP to RR), and synergistic (TE from RESP and SAP to RR that is not explained by either source alone) contributions. These measures reflect the following physiological mechanisms: the unique contribution from SAP to RR (U_SAP→RR_) quantifies the influence of SAP on RR independent of RESP, linked to the cardiac chronotropic baroreflex; the unique contribution from RESP to RR (U_RESP→RR_) highlights the non-baroreflex, mostly central effects of RESP on RR; the redundant contribution (R_RESP,SAP→RR_) describes the baroreflex-mediated effect of respiration on heart rate, quantifying the contribution of the indirect pathway RESP→SAP→RR; and the synergistic contribution which describing simultaneous presence of baroreflex and non-baroreflex respiratory influences (S_RESP,SAP→RR_).

The multiscale PID approach enables computation of all relevant information measures at any chosen time scale (τ) directly from the coefficients of the vector autoregressive (VAR) process fitted to the time series. In this study, VAR models were identified for RESP, SAP, and RR using the least squares method, with model order selected also by the Akaike Information Criterion. To capture fast respiratory and baroreflex effects, the model was extended to include zero-lag influences from RESP to SAP and RR, and from SAP to RR.

Since interactions between cardiovascular and respiratory time series are predominantly reflected at shorter time scales, we specifically performed the analysis using a time scale of τ_1_ = 1 (unique TE from RESP to RR, redundant TE from RESP through SAP to RR, and synergistic TE from RESP and SAP to RR), covering all oscillations. In contrast, selected PID measure at a longer time scale (τ_2_), which removes HF oscillations and focuses on slower oscillations, was used for characterization of the overall involvement of baroreflex on RR oscillations – unique TE from SAP to RR. The scale τ_2_ determined individually for each subject and experimental condition as:

$$\tau_{2}=\frac{1}{2f_{\tau }\bar{RR}},$$

where *f* *_τ_* is the cut-off frequency of the low-pass filter (0.15 Hz) and $\bar{RR}$ is the mean RR interval in seconds. Further methodological details are available in Krohova et al. (2019) and Faes et al. (2017).

### 2.3 Statistical analysis

Group comparisons between controls and patients with PD, considering Gaussian or non-Gaussian data distribution, were performed using Student’s t-test or Mann-Whitney test, respectively, for each measure. PD subgroups comparison (GIT+ vs. GIT-) was performed using the Mann-Whitney test. Results were considered statistically significant at P-values <0.05. Statistical analysis was performed using SYSTAT 13 (Systat Software Inc., United States).

## 3 Results

Differences in the mean/median values of the cardiovascular and respiratory time series are presented in Table 1. In the evaluated PD and C groups, we did not find significant differences in mean values of RR, SAP, and tidal volume during whole protocol (P ≥ 0.309). In contrast, respiratory rate was significantly higher in PD throughout the entire protocol (P ≤ 0.038).

**TABLE 1:** Differences in the mean/median values of observed cardiovascular and respiratory parameters between PD and C groups.

Parameters	Supine rest		HUT		Supine recovery	
	PD	C	PD	C	PD	C
Mean RR [ms]	855.3 (81.7)	875.5 (156.4)	748.6 (80.9)	804.7 (173.6)	907.3 ± 144.6	956.2 ± 124.2
Mean SAP [mmHg]	133 ± 13.5	128.8 ± 12.5	129.5 ± 12.2	125.6 ± 14.0	134.4 ± 14.4	131.4 ± 13.4
Median respiratory rate [Hz]	**0.32** ± **0.05**	**0.28** ± **0.04**	**0.31** ± **0.04**	**0.28** ± **0.03**	**0.31** ± **0.05**	**0.28** ± **0.04**
Median tidal volume [L]	0.36 ± 0.13	0.36 ± 0.16	0.38 ± 0.13	0.36 ± 0.16	0.35 ± 0.13	0.33 ± 0.15

Values are presented as mean ± standard deviation for normally distributed variables, median and interquartile range (IQR) for non-normally distributed variables. Statistically significant differences between groups are indicated in bold. Abbreviations: C, control group; PD, PD, patients; SAP, systolic blood pressure; RR, R-R interval.

**FIGURE 1:**
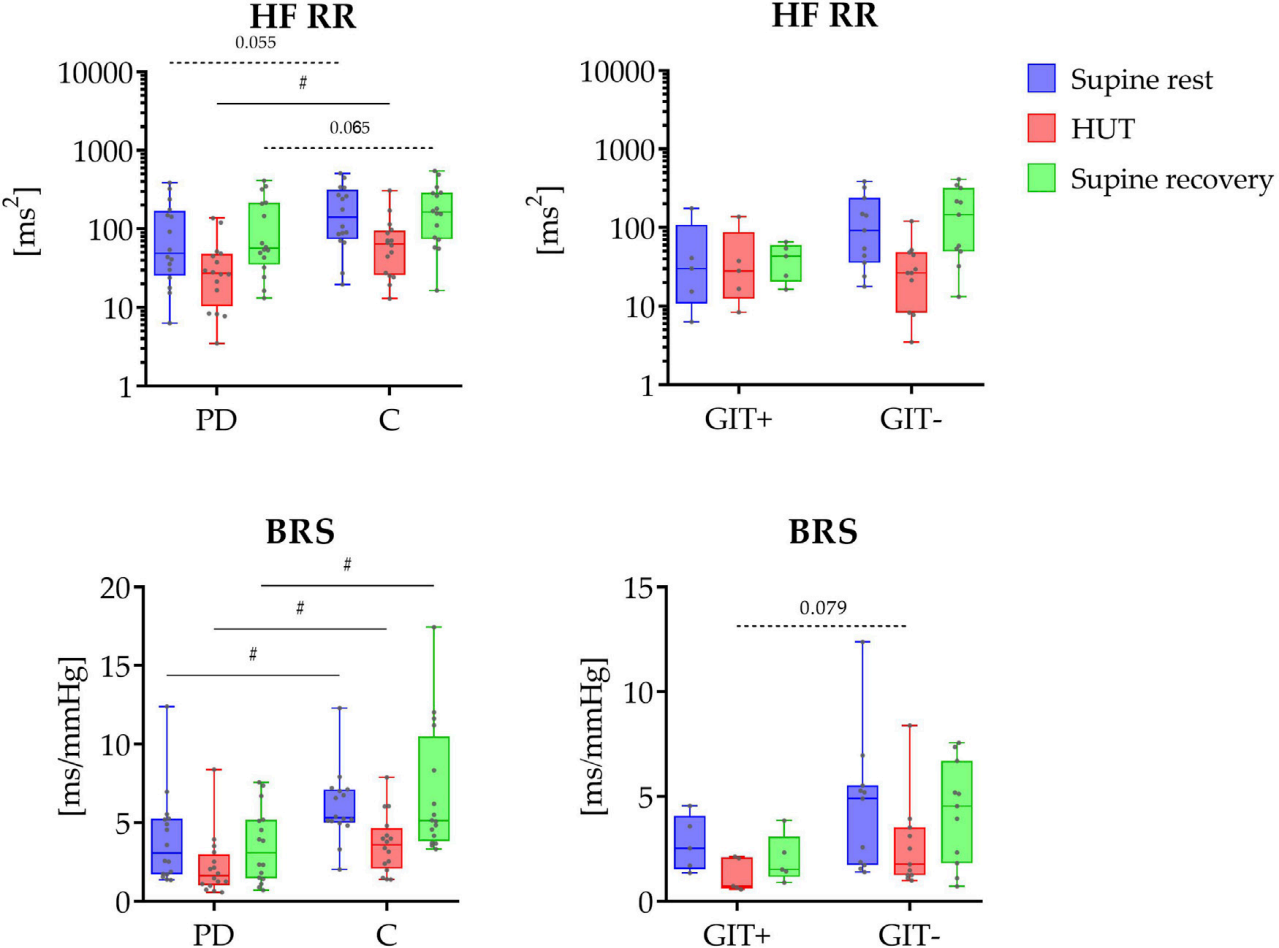
Top panel shows the distribution of HRV spectral power values in the HF band (HF RR; y-axis in logarithmic scale) across three phases (supine rest, HUT, and supine recovery). The bottom panel displays the distribution of causal baroreflex sensitivity during study protocol. On the left side, data are presented for PD patients (PD) and healthy controls (C); on the right side, for PD patients with (GIT+) and without (GIT-) the presence of constipation. The data distributions are shown as box plots and individual values. The box plots report minimum and maximum values (whiskers) and (25, 50, 75)th percentiles (box with central line). # indicates a statistically significant difference between the groups.

The results of spectral HRV analysis and BRS analysis are shown in Figure 1. Spectral analysis revealed significantly lower spectral power in HF band during HUT (P = 0.038) in PD patients compared to control group, with a similar trend observed during the rest phases (P = 0.055 and P = 0.065). No significant differences were observed between gastrointestinal issues-positive and -negative subgroups (P ≥ 0.100 for GIT+ vs. GIT- comparison).

Causal baroreflex analysis revealed significantly reduced coupling gain (BRS) throughout the study protocol in PD patients (P ≤ 0.014), with no significant differences between constipation subgroups (P ≥ 0.079).

**FIGURE 2:**
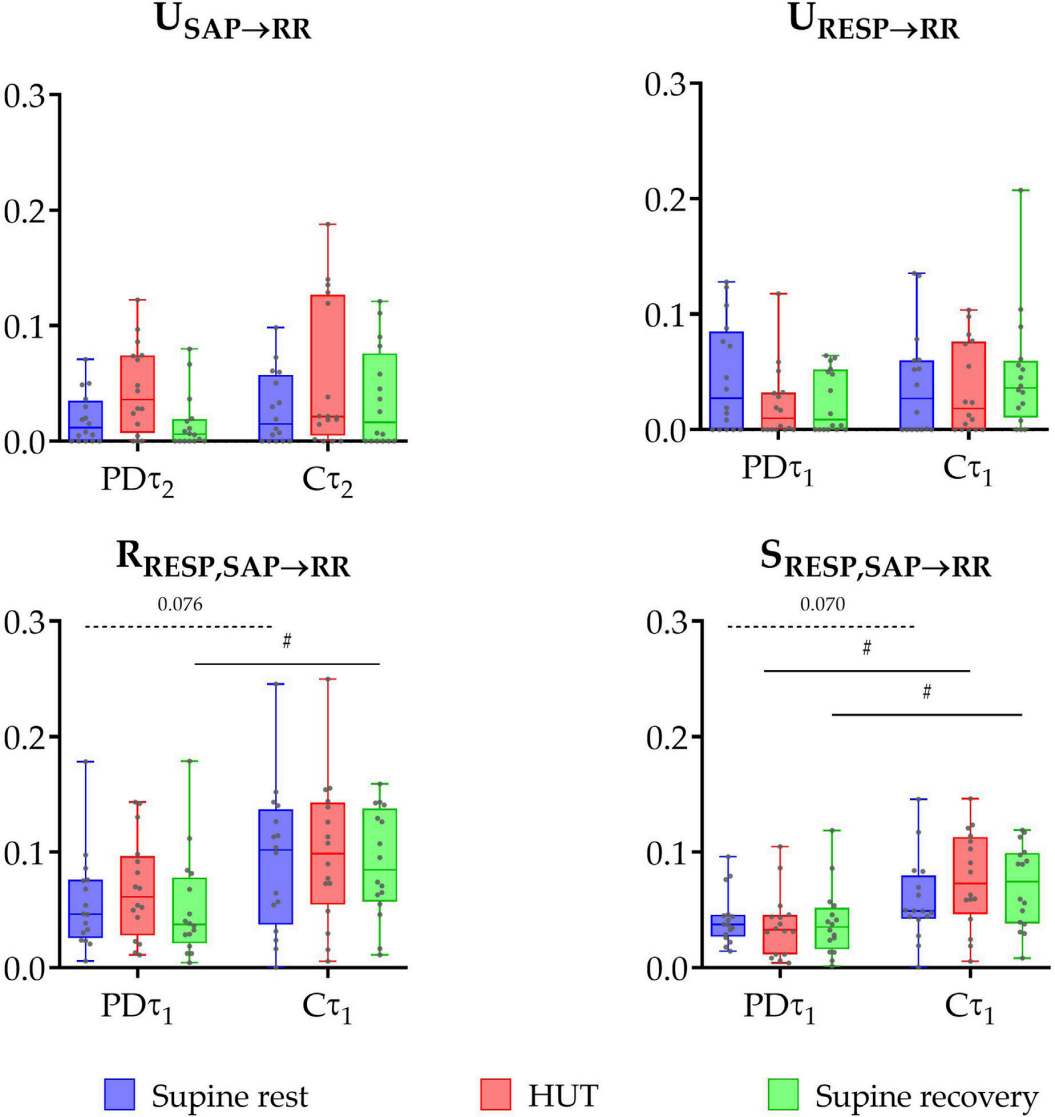
Box plot distributions (reporting minimum and maximum values (whiskers) and (25, 50, 75)th percentiles (box with central line)) and individual values of unique transfer entropy from SAP to RR (U_SAP→RR_) and from RESP to RR (U_RESP→RR_), redundant (R_RESP,SAP→RR_), and synergistic (S_RESP,SAP→RR_) transfer entropy during three phases of the protocol (supine rest, HUT, and supine recovery) calculated for the raw (non-filtered) data (τ_1_) and slower oscillations (τ_2_) for the group of PD patients (PD) and healthy controls (C). Unique TE from SAP to RR was calculated at τ_2_ (capturing slower oscillations within the very low and low frequency bands), while other measures — unique TE from RESP to RR, redundant and synergistic TEs — were calculated at τ_1_ (representing the raw data). # indicates a statistically significant difference between the group of PD and healthy subjects.

The results of information domain analysis are shown in Figure 2. The comparison between early-diagnosed PD patients and control group did not find any significant differences in the unique TEs from RESP to RR (P ≥ 0.209) or from SAP to RR (P ≥ 0.265). In contrast, the PD group exhibited significantly lower values of redundant TE during supine recovery (P = 0.022), with a similar trend in supine rest phase (P = 0.076) in the raw data. Synergistic TE was also significantly lower in PD patients during most phases (HUT and supine recovery, P ≤ 0.013), with a tendency of persisting lower values during supine rest (P = 0.070).

**FIGURE 3:**
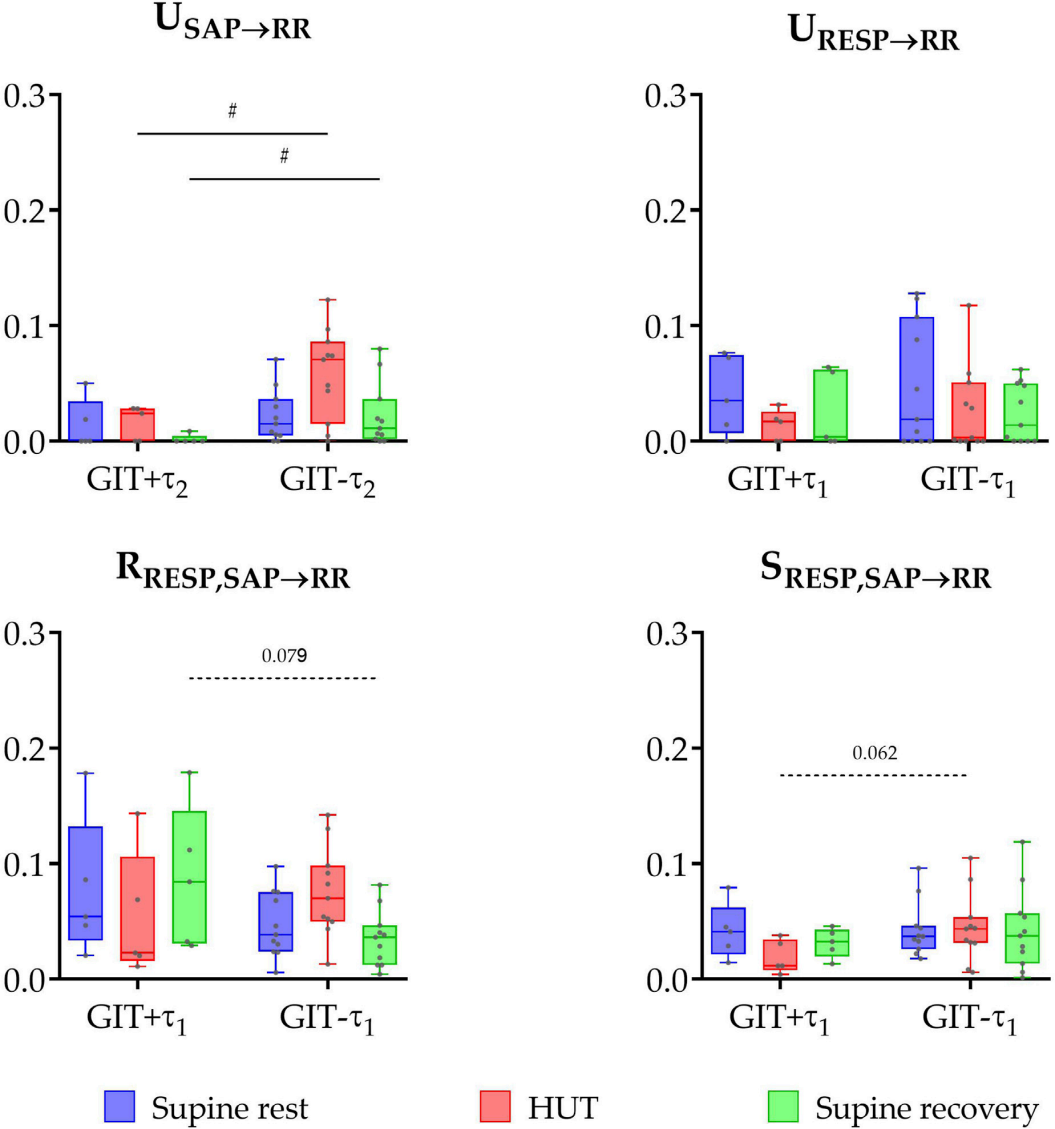
Box plots (showing minimum and maximum values as whiskers, the 25th, 50th, and 75th percentiles as box with central line) together with individual values of unique transfer entropy from SAP to RR (U_SAP→RR_) and from RESP to RR (U_RESP→RR_), redundant (R_RESP,SAP→RR_), and synergistic (S_RESP,SAP→RR_) transfer entropy during three phases of the protocol (supine rest, HUT, and supine recovery) for the group of PD patients with (GIT+) and without (GIT-) the presence of gastrointestinal issues (constipation). # indicates a statistically significant difference between the groups.

Significant group differences in information domain measures (Figure 3) were also observed based on constipation status (GIT+ vs. GIT- comparison). Specifically, unique TE from SAP to RR was significantly lower in the group with gastrointestinal issues during both HUT and supine recovery phases (P ≤ 0.047). No significant differences were found in unique TE from RESP to RR and redundant TE across all phases of the protocol (P > 0.062). However, some trends were observed: the constipation-negative group (GIT-) tended to have lower values of redundant TE during the supine recovery phase (P = 0.079) and higher values of synergistic TE during HUT (P = 0.062).

## 4 Discussion

This study provides a comprehensive, non-invasive analysis of cardiovascular PNS function in the early-stage of PD (less than 6 months after the motor symptoms occurrence) using both conventional and advanced analytical methods. The key finding is that causal evaluation of BRS revealed consistently lower values in PD patients across all phases of the protocol. Furthermore, an advanced information-theoretic method (PID), which allowed us to separate baroreflex-mediated from non-baroreflex contributions to RespHRV, showed a specific reduction in the RESP→SAP→RR coupling pathway in PD patients, indicating early dysfunction of the cardiac chronotropic baroreflex. In parallel, using traditional HRV analysis, patients with early-stage PD exhibited significantly lower spectral power in the HF band during orthostatic challenge, indicating reduced parasympathetic activity. Additionally, stratification of the PD cohort based on the presence of constipation showed that baroreflex function was more severely impaired in the group with gastrointestinal issues (constipation), consistent with the ‘body-first’ subtype of PD.

Previous studies on PD patients with a longer duration of disease demonstrated alterations in HRV indices observed in time and frequency domain, which are under the dominant parasympathetic control. Parameters such as spectral power in the HF band, time-domain indices like CVRR (coefficient of variation of RR intervals) (Suzuki et al., 2017), RMSSD (the root-mean-square of successive beat-to-beat differences in R-R interval duration), and pNN50 (percentage of pairs of successive normal-to-normal (NN) R-R intervals differing in their length by more than 50 milliseconds) were also found significantly reduced in PD patients compared to healthy controls (Haapaniemi et al., 2001; Devos et al., 2003; Maetzler et al., 2015; Perez et al., 2015; Vianna et al., 2016). In our study comparing PD patients less than 6 months after the first occurrence of motor symptoms with age- and sex-matched healthy controls, we found significantly lower spectral power of RR intervals in HF band (the overall magnitude of RespHRV) during orthostatic challenge. A tendency toward reduced values was also observed during the supine rest phases. These findings align with previous studies by Mihci et al. (2006) and Soares et al. (2013), which consistently demonstrated significantly reduced HF power in PD patients. These previous studies conducted in patients with a mean disease duration of around 6 years and regardless of medication status (pharmacological treatment was not interrupted), support the presence of impaired parasympathetic activity under both baseline and postural stress conditions. Our findings extend this evidence by demonstrating a parasympathetic impairment reflected by a decreased RespHRV magnitude already in the early-stage of PD during more enhanced protocol involving three phases. However, some studies found no significant differences in HRV indices between PD patients and healthy controls in time and frequency domain (Ke et al., 2017; Kim et al., 2016; Oka et al., 2011; Vianna et al., 2016; Barbic et al., 2007; Friedrich et al., 2008; Oleksakova et al., 2023), highlighting heterogeneity in autonomic dysfunction, possibly due to differences in PD subtypes, disease duration, or medication status. These findings underscore the complexity of autonomic impairment in PD and the importance of combining HRV analysis with more advanced methods to better characterize parasympathetic control impairment.

Using frequency-domain HRV analysis, we obtained information about the overall magnitude of RespHRV. To gain deeper insight into the physiological mechanisms underlying the above-reported results, we additionally applied the information-theoretic analysis – specifically the multiscale PID. This allowed us to non-invasively distinguish between two mechanisms through which respiration influences heart rate variability (RespHRV): (1) baroreflex-mediated respiratory effects on heart rate oscillations (an indirect pathway: RESP→SAP→RR) and (2) non-baroreflex direct effects unrelated to SAP oscillations (dominantly of central origin resulting from the interconnection between cardiovascular and respiratory centers; a direct pathway RESP→RR). At rest – recovery supine rest phase after HUT – a lower contribution of baroreflex RespHRV mechanisms was found in the PD group, as reflected by the lower redundancy between influences of RESP and SAP on RR (R_RESP, SAP→RR_) in comparison with controls. A similar trend was also observed in the first rest phase – supine rest phase before HUT. A decreased redundancy indicates that the indirect connection between RESP and HRV – cascade RESP→SAP→RR – is partially suppressed in the PD group compared to controls. It could reflect the initial impairment of cardiac chronotropic baroreflex function in this group. Importantly, the synergistic contribution (RESP + SAP→RR), which describes the simultaneous presence of baroreflex and non-baroreflex respiratory influences on RR, was also significantly reduced in PD patients during HUT and supine recovery, with a tendency toward lower values already during supine rest. Together, these results indicate that both redundant and synergistic respiratory contributions to HRV are diminished in early PD, highlighting a weakening of complementary autonomic mechanisms that normally stabilize cardiorespiratory parameters.

In previous studies using PID involving healthy young participants and young obese individuals (median age ∼17 years), redundancy significantly increased during HUT (Javorka et al., 2020; Krohova et al., 2018), indicating that postural stress induces an activation of the baroreflex, resulting in its higher involvement in the RespHRV origin. A similar trend was observed in our PD patients. However, this response was not evident in our control group (median age 56.1 years), suggesting that age-related autonomic changes may blunt this modulation of RespHRV mechanisms in response to postural challenge.

The comprehensive picture of non-invasive evaluation of PNS activity in PD patients is further complemented by the assessment of baroreflex function. The baroreflex is the primary cardiovascular reflex mechanism responsible for short-term regulation of blood pressure, typically representing reflexive vagally mediated responses of heart rate to blood pressure fluctuations. Its sensitivity (BRS) is usually quantified by analysing spontaneous oscillations in SAP and RR intervals, using non-causal approaches based on an assumption of a unidirectional influence from SAP to RR (Pospíšil et al., 2010; Sabino-Carvalho et al., 2020).

In interpreting the information-domain results, it is important to consider the influence of respiratory pattern on cardiorespiratory interactions. Although no significant group differences were observed in tidal volume across the protocol, patients with early PD consistently exhibited higher breathing frequency compared to controls. This increase is likely multifactorial – primarily driven by chest wall rigidity and reduced thoracic compliance, but also influenced by central respiratory control deficits, upper airway dysfunction, and autonomic dysregulation (McMahon et al., 2023). Breathing frequency is one of the major determinants of RespHRV magnitude: faster breathing shortens the respiratory cycle and reduces the amplitude of RespHRV, even in the absence of vagal tone changes (Elghozi et al., 1991; Larsen et al., 2010). Therefore, the interpretation of decreased HF power in PD patients as a parasympathetic withdrawal should be taken with caution. Since information transfer reflects the engagement of RespHRV-related mechanisms, elevated breathing frequency likely contributed to the observed patterns: faster respiration shifts cardiorespiratory oscillations to higher frequencies, reducing the effectiveness of vagal modulation and weakening the transmission of respiratory influences into RR variability. This mechanism may help to explain the reduced redundancy and synergy, as higher respiratory frequency alters the cooperative and overlapping contributions of respiration and blood pressure to RR.

In PD patients with a longer disease duration – typically more than 5 years – several studies have reported significantly reduced BRS compared to healthy controls during supine rest, indicating a decline in parasympathetic responsiveness. These findings were generally based on non-causal assessment (Pospíšil et al., 2010; Sabino-Carvalho et al., 2020). Similarly, Blaho et al. (2017) found a reduced BRS in PD patients (mean duration 6.7 ± 3.9 years) with the reduction being particularly pronounced in those presenting with orthostatic hypotension, further underscoring the clinical importance of BRS assessment in this population.

In contrast, findings in early-stage PD patients have been more variable. Oka et al. (2011), examining patients within 3 years of symptom onset, found no significant differences in BRS between PD patients and controls. Our current results extend these observations even further by assessing BRS using causal method, which is more precise (less influenced by non-baroreflex mechanisms) compared to non-causal approaches (Porta et al., 2002), in patients within 6 months of motor symptom onset. Consistent with previous findings, we observed a significant reduction in BRS throughout the entire protocol, suggesting an early functional impairment of the baroreflex pathway. Additionally, the use of a PID-derived parameter – unique TE from SAP to RR – offered complementary insight into baroreflex function. Interestingly, this measure did not show significant differences across phases, suggesting that the coupling strength between SAP and RR oscillations – in contrast to BRS – remains relatively preserved in very early PD.

From the perspective of optimizing early diagnosis and treatment of PD, research over the past two decades has focused on improving our understanding of the mechanisms involved in its onset and progression. One of the most probable mechanisms of PD development is the intraneuronal accumulation of pathologically altered α-synuclein protein, which is capable of spreading via a so-called “prion-like” mechanism along neural pathways from one centre to another (Jain, 2011). Traditional models, such as Braak’s hypothesis, suggest that neurodegeneration begins in the enteric nervous system and spreads via the vagus nerve to the brainstem, substantia nigra, and higher centers within the central nervous system (Braak et al., 2004).

Expanding on this concept, the α-Synuclein Origin site and Connectome (SOC) model proposes two distinct subtypes of PD based on the anatomical origin of neurodegeneration: the “body-first” and “brain-first” subtypes (Borghammer, 2021). In the “body-first” subtype, pathology originates in the peripheral autonomic nervous system – e.g., in the gastrointestinal tract – and ascends to the brain via the vagus nerve. This form is associated with an early autonomic dysfunction, including symptoms like REM sleep behaviour disorder, hyposmia, and constipation, as well as more symmetric motor involvement and faster progression of the non-motor symptoms. In contrast, the “brain-first” subtype originates within central structures including the amygdala or locus coeruleus and spreads downward to the brainstem and peripheral nervous system. This form is typically associated with asymmetric motor symptoms, slower disease progression, and fewer early autonomic or non-motor symptoms. The SOC model also suggests that the prodromal phase is shorter in “brain-first” subtype due to direct, monosynaptic connections between the origin site and the substantia nigra (Borghammer, 2021).

Building on this framework, changes in PNS activity – including those reflected in HRV, BRS, or autonomic impairment symptoms like constipation – may be more pronounced in individuals with peripheral-onset/“body-first” PD subgroup. In our study, we stratified the PD group based on the presence of gastrointestinal issues (constipation) and observed significant group differences in parameters reflecting PNS activity. Specifically, unique TE from SAP to RR reflecting involvement of baroreflex in heart rate dynamics was significantly lower in the group with the presence of gastrointestinal issues during both the head-up tilt and supine recovery phases. These findings support the SOC model and suggest that the “body-first” PD subtype may be characterized by earlier and more marked ANS impairment.

As noted, our PD cohort likely included a higher proportion of “brain-first” cases compared to “body-first,” which may have significantly influenced the observed results. This imbalance could also help to explain some of the discrepancies in previous studies focusing on autonomic dysfunction in PD, as variations in the relative distribution of these two fundamental subtypes across different study populations may partially account for inconsistencies in reported findings.

Overall, the body of evidence, including our results, indicates that parasympathetic dysfunction is a measurable and likely progressive phenomenon that begins early in the course of PD. This is detectable not only through conventional methods, e.g., HRV analysis and BRS assessment, but also through more sophisticated information-theoretic approaches offering an enhanced insight into underlying regulatory mechanisms. Our findings are consistent with previous reports showing reduced parasympathetic function in the early (Pursiainen et al., 2002) and even prodromal stages of PD (Alonso et al., 2015).

However, variability across studies on autonomic dysfunction in PD still remains an ongoing challenge. Differences in methodology, patient characteristics, and analytical techniques may account for inconsistency of findings. The heterogeneity of PD itself, especially the existence of distinct clinical subtypes (e.g., “body-first” vs “brain-first”), likely also contributes to differing presentations of autonomic dysfunction. These results highlight the need for future longitudinal studies employing multimodal and sensitive analytic tools to better characterize the trajectory of parasympathetic impairment and its potential role in early diagnosis, disease stratification, and progression monitoring in PD.

Our work demonstrates how advanced signal processing and network physiology-based methods can detect parasympathetic dysfunction in early PD. By analysing cardiorespiratory and cardiovascular interactions through techniques such as PID, we move beyond single-variable analyses (e.g., HRV analysis in frequency domain) to examine how multiple subsystems interact dynamically, revealing alterations in redundancy and compensatory mechanisms. Future studies integrating additional networks, including the gut-brain axis, could further clarify systemic mechanisms in PD and support more comprehensive assessments of physiological interactions.

## 5 Conclusion

This study provides compelling evidence for an early PNS dysfunction in PD, less than 6 months after motor symptom onset. While conventional HRV parameters such as spectral power of HF band was significantly reduced during orthostatic stress, PID offered additional mechanistic insights by separating baroreflex-mediated and non-baroreflex contributions to RespHRV. The reduced redundancy between SAP and RESP inputs to RR (RESP→SAP→RR coupling pathway) in PD patients further suggests an early impairment of the cardiac chronotropic baroreflex mechanism.

Stratifying patients by the presence of constipation – a hallmark of the “body-first” PD subtype – revealed more pronounced baroreflex dysfunction in this group, lending support to the SOC model. These findings emphasize the heterogeneity of autonomic impairment in PD and suggest that early non-invasive assessment of vagal function can help to distinguish PD subtypes.

## 6 Study limitations

One of the main limitations of this study is the relatively small sample size, primarily due to the strict inclusion criteria requiring the enrolment of PD patients within 6 months of motor symptom onset. While this approach increases the specificity of findings related to early-stage disease, it inherently reduces statistical power and raises the risk of Type II error, potentially obscuring subtler effects. Nevertheless, despite this limitation, multiple analytical methods–including both conventional and information-theoretic approaches–revealed significant differences, particularly indicating early cardiovascular baroreflex dysregulation in this patient group.

Another important limitation is an uneven distribution of PD subtypes within the evaluated cohort. Our findings suggest a predominance of the “brain-first” subtype among the included participants, which may have influenced the observed patterns of autonomic dysfunction. This imbalance limits the generalizability of the results to the broader PD population and underscores the need for future studies with larger, more balanced samples.

## Data Availability

The raw data supporting the conclusions of this article will be made available by the authors, without undue reservation.
